# Supplementary material for: From the Lab to the Field: Long-Distance Transport of Sterile Aedes Mosquitoes
Source: Insects. 2023 Feb 18;14(2):207. doi: 10.3390/insects14020207 (PMC9967802; doi:10.3390/insects14020207)
Supplement: Supplementary file 1 [file insects-14-00207-s001.zip › Supplementary File S1_Detailed protocol for packing and shipping sterile male Aedes mosquitoes.pdf]

DETAILED PROTOCOL FOR PACKING AND SHIPPING STERILE MALE *AEDES* MOSQUITOES

Version 1.0

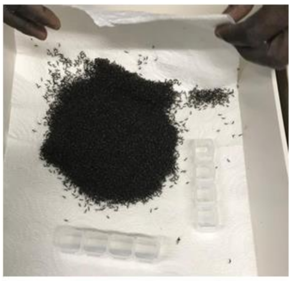
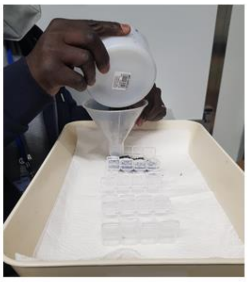
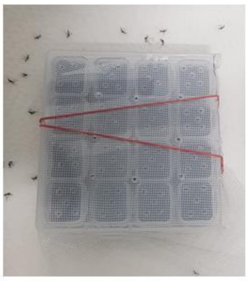
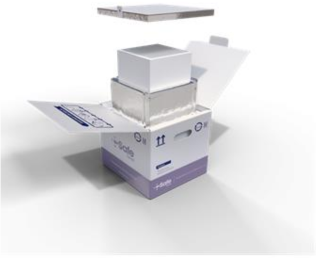
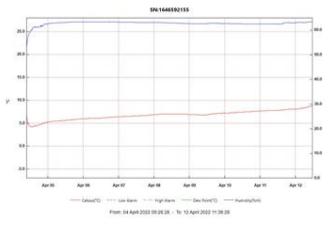
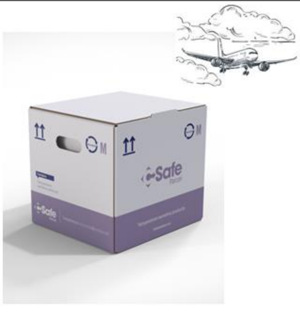

Edited by:

Hamidou Maiga, Hanano Yamada, and Jeremy Bouyer

Cover photo credit: Hamidou Maiga and Thomas Wallner

DISCLAIMER

The mention of specific companies or a certain manufacturers' products in this document does not imply that they are endorsed or recommended in preference to others of a similar nature that are not mentioned.

Contents

1. Introduction 4
2. Materials 4
3. Chilling, Compaction, and Irradiation procedures 8
4. Mosquito packaging procedures 11
5. References 18
6. Acknowledgments 18
7. Appendix 18

1. Introduction

Pilot programmes of the sterile insect technique (SIT) against *Aedes aegypti* may rely on importing significant and consistent numbers of high-quality sterile males from a distant mass-rearing factory. As such, long-distance mass-transport of sterile males may contribute to meet this requirement if their survival and quality are not compromised.

The described system allows to mass-transport sterile male *Aedes* mosquitoes by air/ground using courier services or air freight without having a significant negative impact on their quality in terms of physical damage, flight ability and survival following transport for up to three days. It was validated through trials of approximately 15 shipments (batches between 10 000-50 000 each) carrying in total more than 400,000 sterile males from Seibersdorf, Austria to Reunion Island and the Republic of Senegal. Three levels of containment are required for their trans-boundary transport to avoid any possible escape[1] (http://www.reunion.gouv.fr/IMG/pdf/2021-282.pdf). The transparent boxes allow routine control activities by customs without any risk of escape.

The following steps and materials are necessary for packing and transporting sterile male *Aedes* mosquitoes.

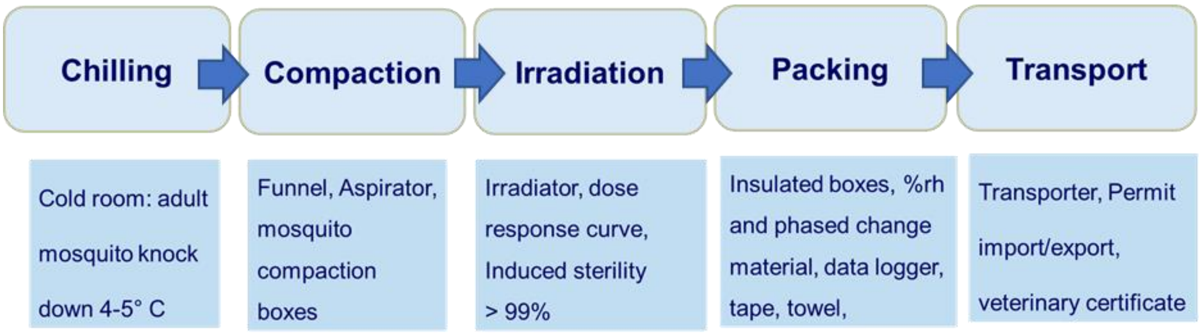

Figure S1. Flowchart equipment and materials for packing and transporting of sterile male *Aedes* mosquitoes

2. Materials

The following materials are required for packaging and shipping sterile male *Aedes* mosquitoes:

- Mosquito compaction boxes (eg DiamondPaintingsx.ch, Switzerland) attached in series of four boxes are used to store the males at a density of 100 males/cm^3 (Figure S2) [4,5]. Each individual box (2.5 × 2.3 × 2.3 cm) has a press down lid that ensures that the lids do not come

off. Each single box contains about 1,300 sterile males. Each individual mosquito compaction box has drilled holes on the top to allow ventilation.

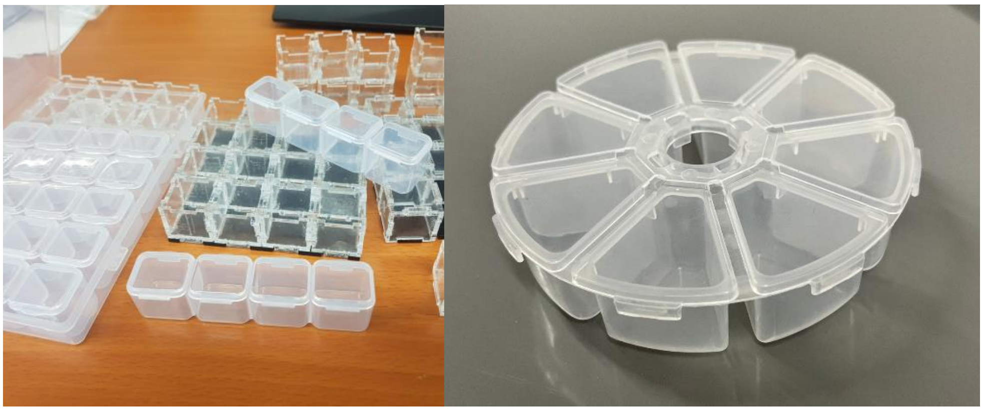

Figure S2. Example of mosquito compaction boxes including Diamond painting boxes, boxes made using a laser cutter 2.2 × 2.2 × 2.2 cm (left) and round type eight compartments V shape individual boxes (height 2.5cm, diameter 10cm) (right)

- Transport data logger (eg MSR175 or equivalent that records Shock, Temperature, Humidity, Pressure and Light) should be placed inside the insulated transport box close to the plastic compaction box to monitor these parameters (https://www.microdaq.com/msr175-shock-pressure-light-humidity-temperature-data-logger.php) (Figure S3)

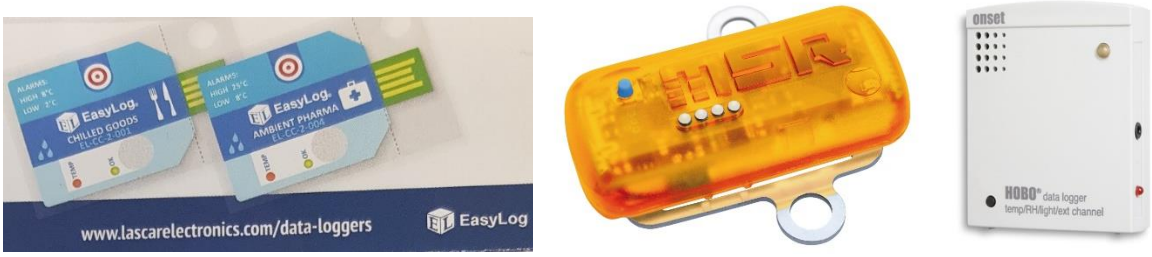

Figure S3. Examples of data loggers that can be used to monitor environmental conditions during shipment/transport of sterile male mosquitoes

- a salt hydrate based Phase Change Material (PCM) pack(s) (eg ClimSel™ C7, 12 × 17 × 1 cm https://www.climator.com/en/pcm-climsel/product-data-sheets) or other PCMs that can maintain temperature between 7 and 12° C during transport[5] are placed in the transport box after at least 3 days of storage at 5°C (Figure S4).

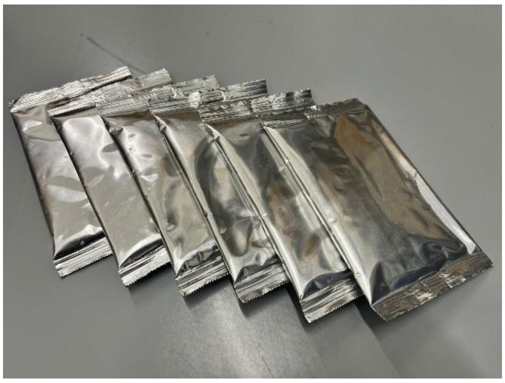

Figure S4. ClimSel™ C7 Phase Change Material packs

- Humidity control packs are also inserted within the box to maintain the relative humidity at 72% (https://bovedainc.com/store/tobacco/boveda-bulk-case/?rh=72) (Figure S5)[5];

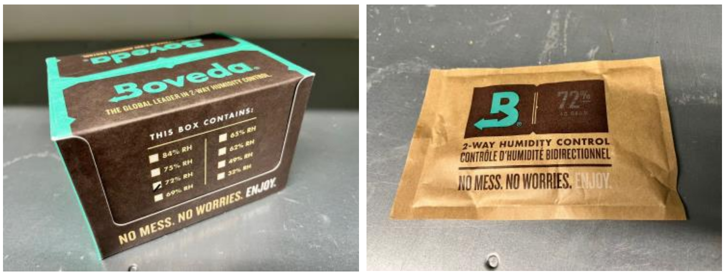

Figure S5. Humidity control packs

- Cotton towels are placed on top of mosquito boxes to maintain the relative humidity and absorb eventual water condensation (Figure S6);

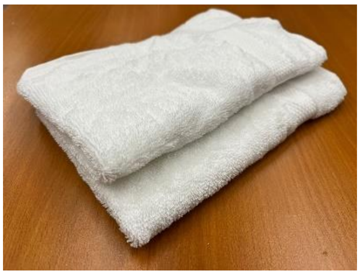

Figure S6. Towels

- Vacuum insulated transport boxes. Two types of insulated boxes are used: A small carton thermobox (inner width × length × height=17.5 × 17.5 × 17.5 cm) (eg Thermoboxes - RAJA (rajapack.at)) is used as an inner carton. The carton is made of 3-5 mm thick extruded polystyrene with metallized polyethylene coating on both sides (Figure S7);

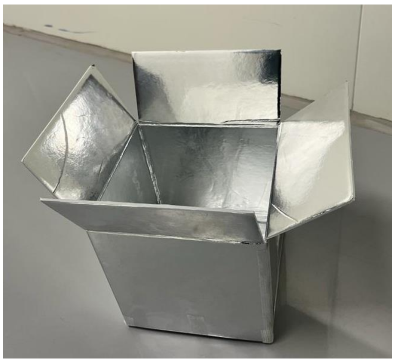

Figure S7. Inner carton made of polystyrene with metallized polyethylene

A bigger carton with inner width × length × height of 17.78 × 17.78 × 17.78 cm and external dimensions of 35.56 × 31.11 × 33.02 cm (eg CSafe Parcel R - 5L - 96hrs, Parcel Solutions - CSafe passive parcel and small active solutions, csafeglobal.com) (Figure S8) .

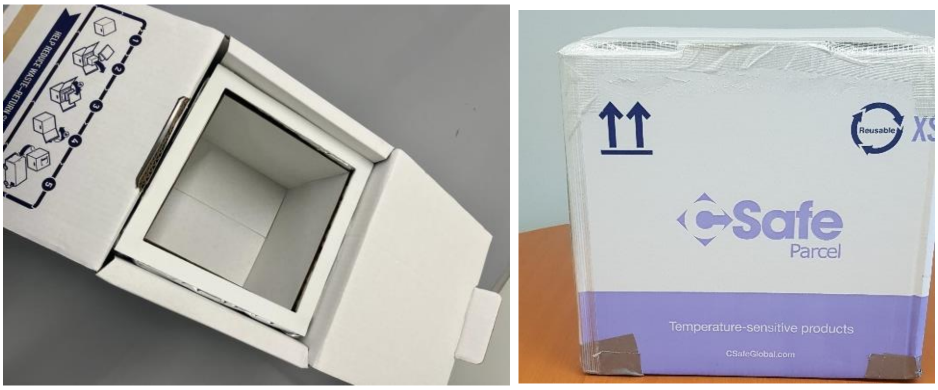

Figure S8. Outer carton inserted in a cardboard external protection (CSafe Parcel R - 5L - 96hrs) (left) and packed ready to ship transport box (right)

- Pieces of Expanded Polystyrene (EPS) with various sizes to hold packs and mosquito compaction boxes inside the inner carton (Figure S9).

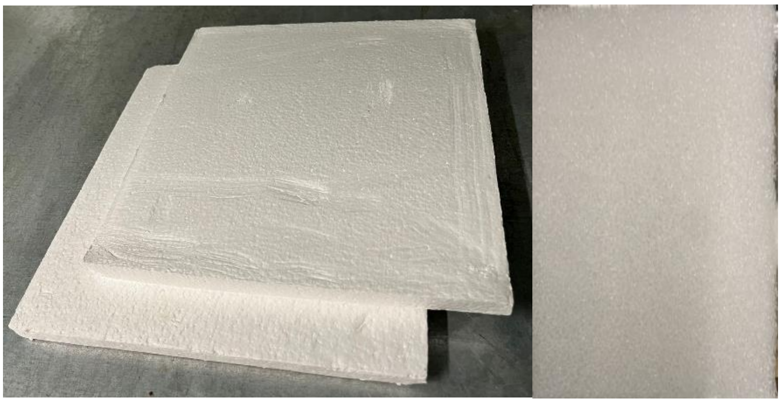

Figure S9. Pieces of Expanded Polystyrene

- Packing EPS peanuts to hold the inner carton within the bigger vacuum insulated box (Figure S8).

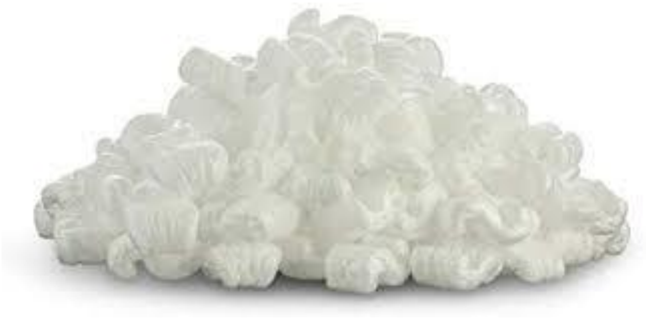

Figure S10. Packing EPS peanuts

- Packing tape (Filament tape - RAJA (rajapack.at) (Figure S11).

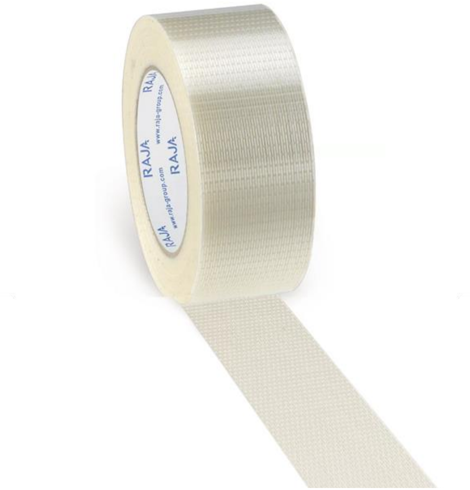

Figure S11. Packing tape

3. Chilling, Compaction, and Irradiation procedures

Remove sugar from the cages (Figure S12) right before chilling mosquitoes in the cold room.. Make sure no sugar or water was spilled inside the cages. To prevent irregular mortality, no

mosquitoes from wet cages were put into the batches to be transported.

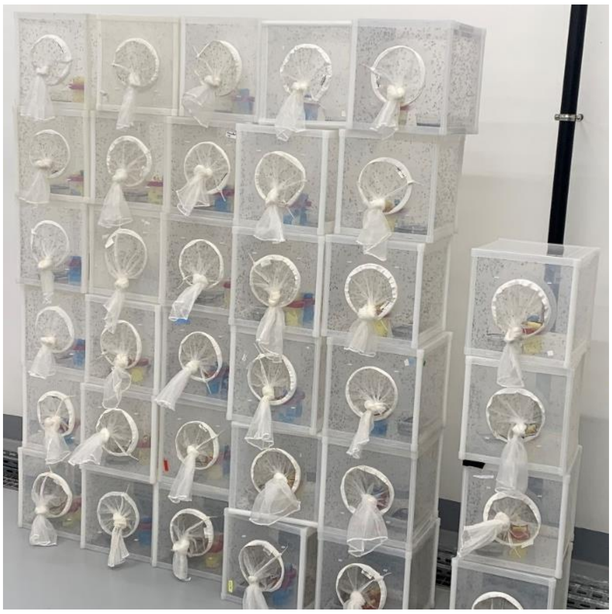

Figure S12. Mosquitoes caged at a density of 2 000 males per 30 x 30 x 30 cm BugDorm cage, maintained under 10% sugar water until the day of packing, irradiation and shipment/transport

- Bring cages into the cold room (4-5° C) and allow a knock down period of 10 min
- Remove mosquitoes from the cage by gently tapping the cages to drop the mosquitoes into a 40 × 30 × 8 cm tray lined with paper towel (to avoid electrostatic interactions) (Figure 13). Stainless steel trays can also be used to avoid electrostatic effects.

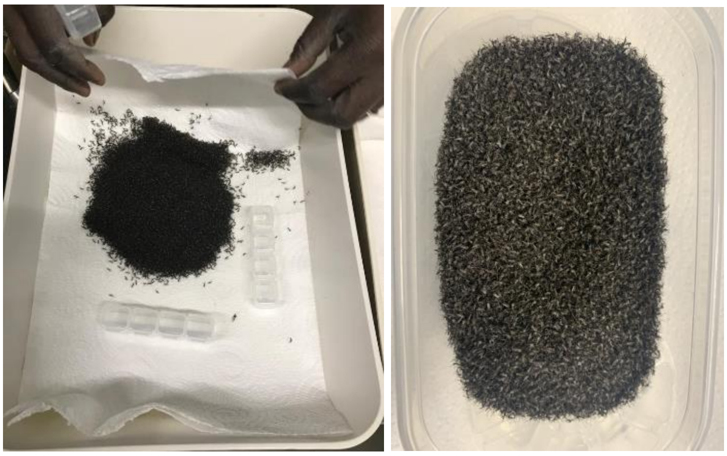

Figure S13. Chilled adult mosquitoes ready to be compacted into mosquito compaction boxes

- Use mosquito compaction boxes (eg. Diamond painting storage boxes) (Figure S14a) and a funnel (Figure S14b), to transfer mosquitoes (Figure S14cd)
- Close each lid (Figure S14ef) and cover with a mosquito net and rubber band (Figure S14g) to avoid escapes. Figure 14 shows material used to chill adults and transfer into mosquito compaction boxes using funnel. Each individual cell can hold up to 1,300 or 1,500 males at a density of 100 males/cm^3 ([4,5]).

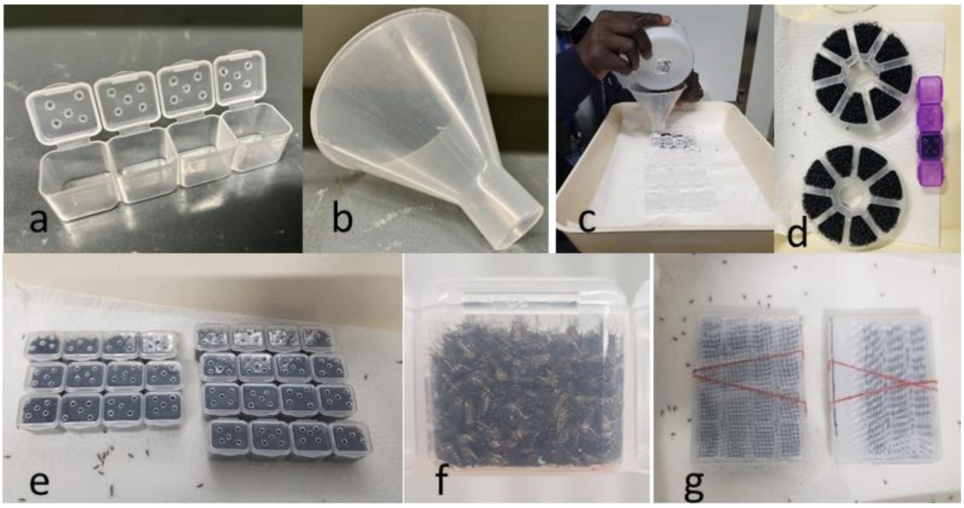

Figure S14. Chilled adults transferred into mosquito compaction boxes using funnel. Each individual cell can hold up to 1,300 or 1,500 males at a density of 100males/cm^3.

- Place mosquito compaction boxes within a larger 11 × 17 cm containment box (individual boxes holder) (Figure S15) with drilled holes to allow air exchange. Mosquitoes are then ready for irradiation.

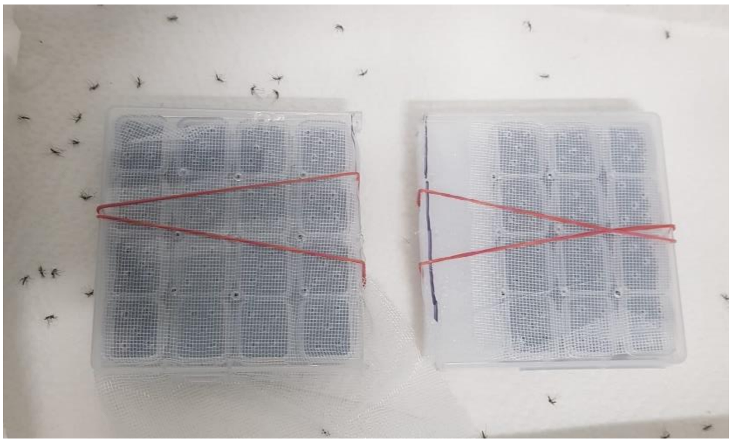

Figure S15. Chilled and compacted mosquitoes ready for irradiation/packing

- Put the larger box into a temperature-controlled transport box (4-8° C) (Figure S16, left). The transport box contains one PCM (eg ClimSel™ C7 pack) at the bottom that is covered with a towel and two PCMs lined along the longest sides of the box. Provide EPS pieces to prevent direct contact between the canister and PCMs. Cover the transport box, seal using tape and transport gently to the irradiation facility. A

Dometic cooling box (https://www.tackledirect.com/dometic-cfx-95dzw-coolfreeze-portable-powered-dual-zone-cooling-box.html) set between 8-10 °C can also be used to transport mosquitoes to the irradiation room/facility. When the irradiator is not close to the rearing facility, consider the section below **(‘4. Mosquito packaging procedures’**) prior to transporting to the irradiation facility.

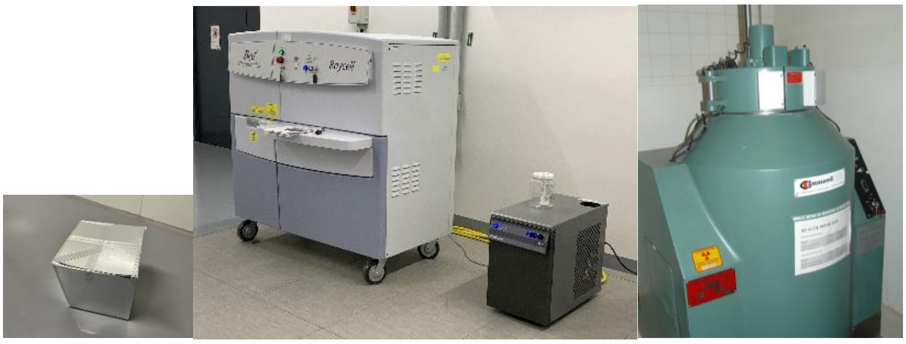

Figure S16. Chilled and compacted mosquitoes (left) ready for irradiation using either an X- (middle) or gamma ray irradiator (right)

- Keep chilling/compaction time to a minimum duration (20-30 min)[5]. Two operators are necessary for about 50,000 males.
- At this phase, mosquitoes are irradiated while maintained in their compaction box. During irradiation the mosquito must be maintained at temperature between 7 and 12 using PCM and appropriate refrigerated container to be introduced in the irradiator machine. For each radiation machine, a dedicate holding refrigerated container must be designed and tested to measure the effective dosimetry to be adopted.
- Once the mosquitoes are irradiated with a dose inducing more than 99% sterility, immediately return the sterile males to the cold room for packaging (4-5 °C for 15 mins/ 50 000 mosquitoes).

4. Mosquito packaging procedures

1 - Place at the bottom of the insulated box: one PCM (eg ClimSel™ C7 pack) and one towel.

2- Place a piece of expanded polystyrene (EPS) on the towel

3- Place one PCM against each of the four sides of the box (four in total), of which two of the PCMs are folded to fit (see Figure S17, right); each is placed at the smaller sides of the box. Folded PCM were conditioned prior to storing in the cold room.

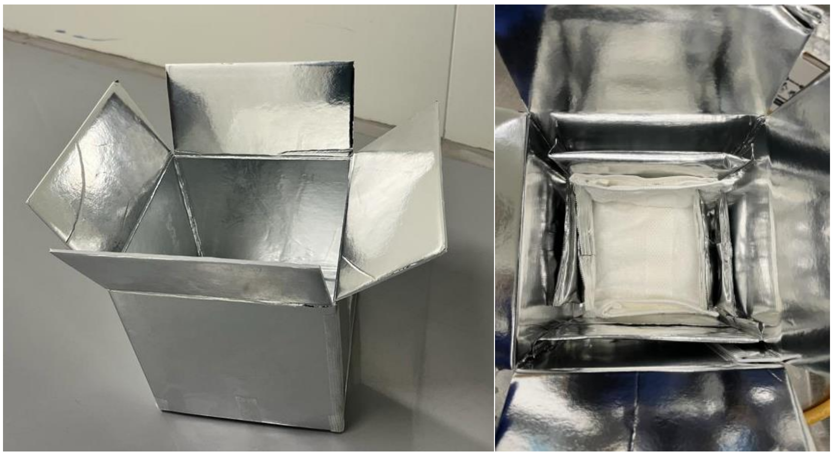

Figure S17. Opened inner carton (left) and towel and PCMs (ClimSel™ C7 packs) lined in (right)

4- Place a small recorder (eg ONSET hobo, recording T°, RH% or a Shock transportation Data Logger (if available), https://www.msr.ch/de/produkt/transport-datenlogger-schock-msr175/) (Figure S18a) in the middle of the EPS (Figure S18b). Add one as a back-up.

5 - Place the plastic box (Figure S18c) containing the sterile male mosquitoes on the piece of EPS. Make sure that the box is not touching the PCMs (Figure S18d) by placing pieces of EPS (Figure S18b) between them and the mosquito box. These pieces of EPS will keep the mosquito box stable during transport. An additional large EPS piece is placed on top of mosquito compaction boxes (Figure S18, right).

6- Place one humidity control packs (72%) (Figure 18e) at each of the sides of the mosquito box.

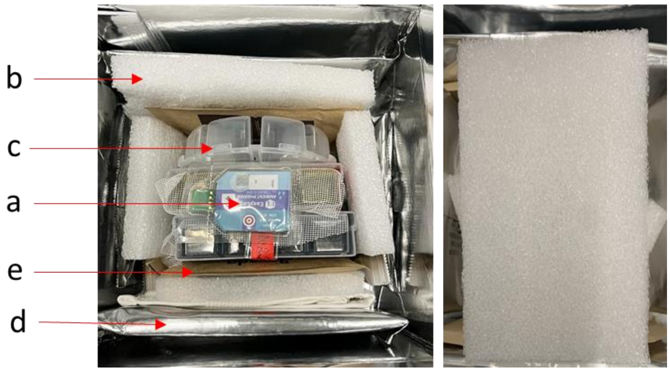

Figure S18. Inner carton containing Hobo logger (a), pieces of Expanded Polystyrene (EPS) (b), mosquito compaction boxes (c), Phased Conditional Material (PCM) (d), Relative humidity packs (e), and a piece of Expanded Polystyrene to put on top (right).

7- Cover with another towel.

8- Place two PCMs (eg ClimSel™ C7 packs) (Figure S19) on top of the towel

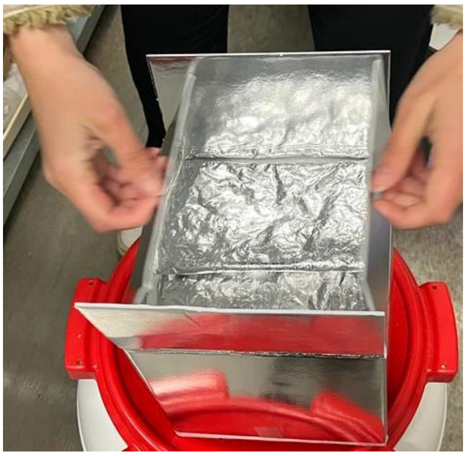

Figure S19. Two PCMs (ClimSel™ C7 packs) on top of the towel in the inner carton.

9- Close the insulated box (inner carton) (Figure S20) with pressure to ensure that the cover fits well, and no air exchange is possible. Seal with tape all around.

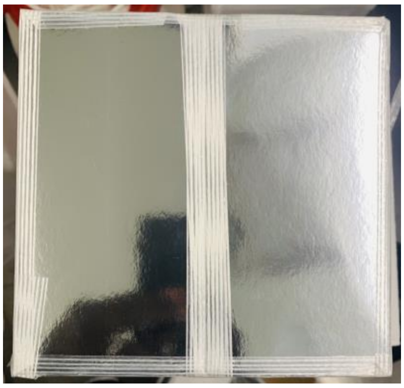

Figure S20. Top of the inner carton sealed with tape.

10. Open the bigger carton (CSafe Parcel R - 5L - 96hrs) (Figure S21, left) and place one PCM at the bottom (Figure S21, right).

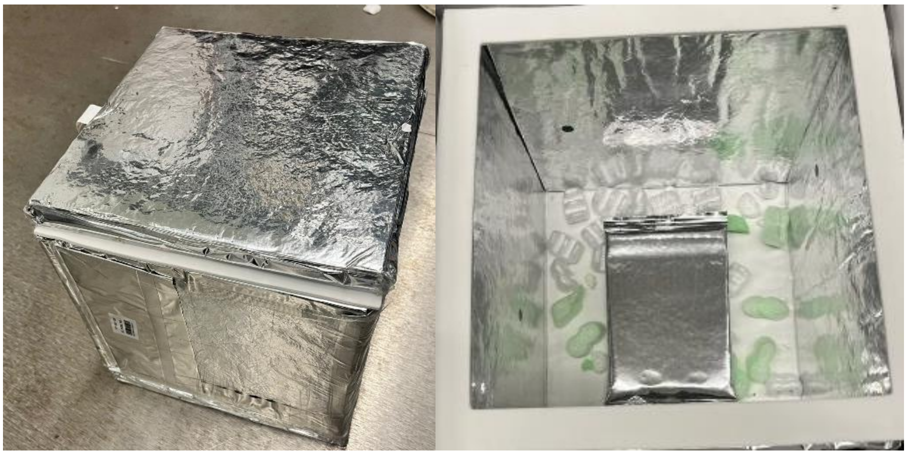

Figure S21. Vacuum insulated box (CSafe Parcel R - 5L - 96hrs) (left) and inside of the outer carton lined with one ClimSel™ C7 pack (right) prior to loading the inner carton.

11. Place the inner carton in the bigger carton (eg CSafe Parcel R - 5L - 96hrs) (Figure S22).
12. Add one PCM at each side of the bigger box (four in total).

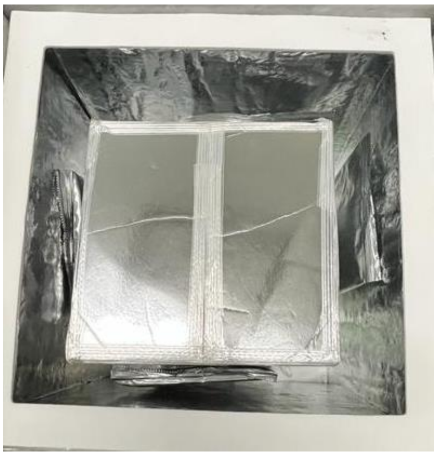

Figure S22. Inside of the outer carton (vacuum insulated box) lined with four ClimSel™ C7 packs around the inner carton.

13. Fill the gaps with packing EPS peanuts to hold the inner carton within the bigger vacuum insulated box (Figure S23).

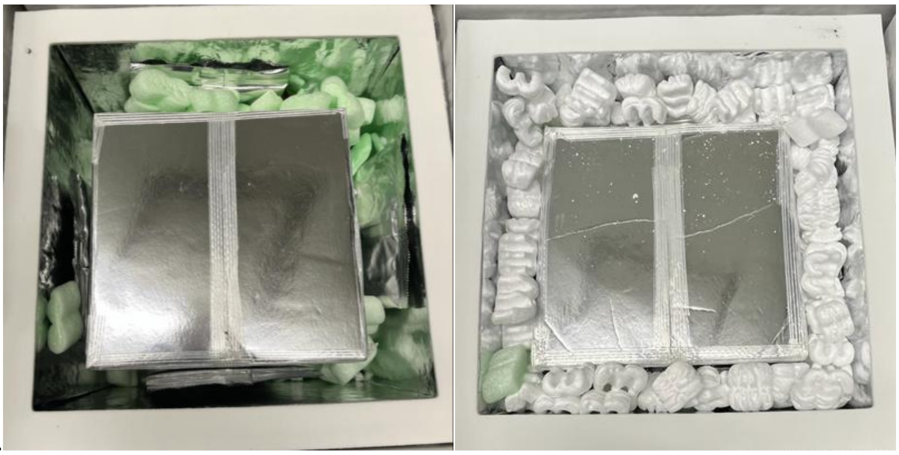
.

Figure S23. Inside of the outer carton filled with packing EPS around the inner carton.

14. Close the bigger carton (vacuum insulated box) (eg. CSafe Parcel R - 5L - 96hrs) with a lid (Figure S24) and seal with tape to ensure that no exchange with ambient air is possible, thus keeping the temperature low inside the box.

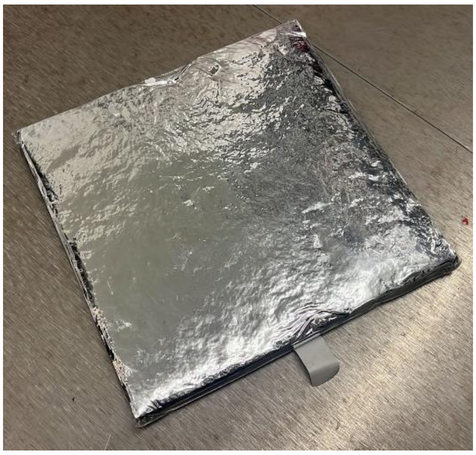

Figure S24. Lid of the outer carton (vacuum insulated box).

15. Book courier pick-up and print the shipping company documents (Figure S25, left) and other required documents and permits including export, import, and certificates confirming absence of health hazard and no commercial value (see appendix). Drop the transport box (Figure S25, right) assembled as in Figure 26, to the pick-up area (procurement office or any appropriate site).

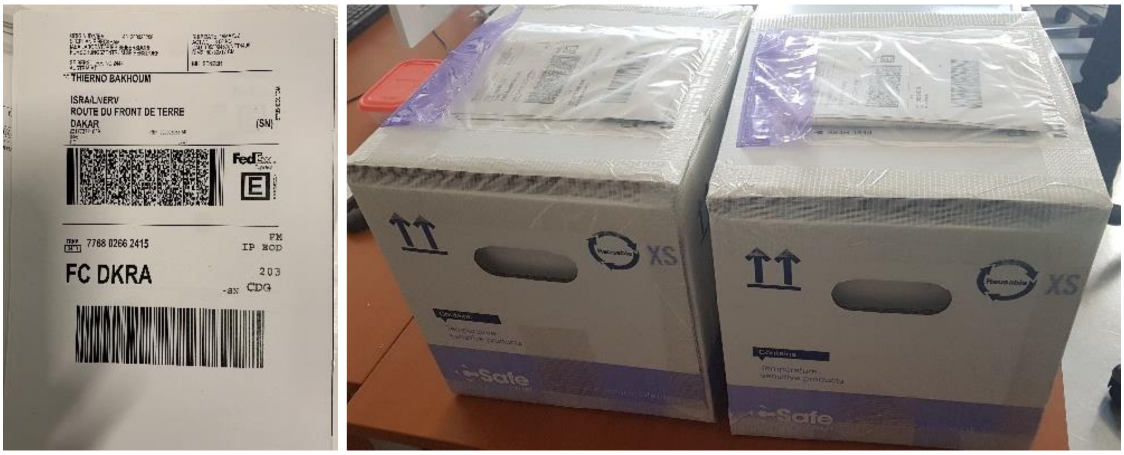

Figure S25. Shipping documents ready (left) and two transport boxes ready to be collected by FedEx (right).

16. Keep track of the shipment and inform the recipient on the status and location.
17. Share the following information including the sterile male age, irradiation date and dose, packaging and shipping time, the payload, size of the package and chilling duration (during chilling and compaction processes) with the receiver/counterpart.
18. Figure S26 shows an assembly of the packing box (mass-transport box (eg CSafe Parcel R -5L - 96hrs)) including the lid (Figure S26A), the inner carton (small carton) (Figure S26B), the outer carton (big carton vacuum insulated box) (Figure S26C) and an external protection (Figure S26D).

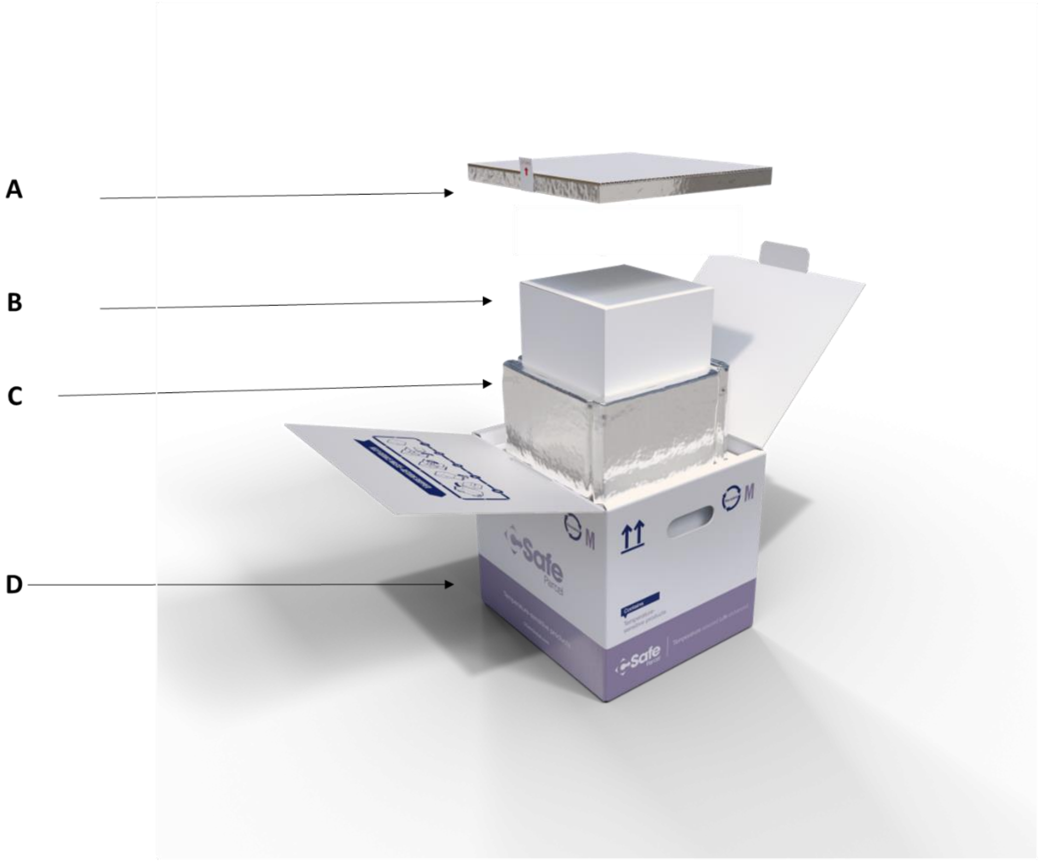

Figure S26. Assembly of the packing box (mass-transport box (eg CSafe Parcel R - 5L - 96hrs)) including the lid (A), the inner carton (small carton) (B), the outer carton (big carton vacuum insulated box) (C) and an external protection (D).

5. References

- [1]FAO/IAEA. (2022). International guideline for transboundary shipments of irradiated sterile insects. Food and Agriculture Organization of the United Nations/International Atomic Energy Agency. Vienna, Austria. 38 pp.
- [2]Culbert NJ, Balestrino F, Dor A, Herranz GS, Yamada H, Wallner T, Bouyer J. A rapid quality control test to foster the development of genetic control in mosquitoes. Sci Rep. 2018 Nov 1;8(1):16179. doi: 10.1038/s41598-018-34469-6. Erratum in: Sci Rep. 2019 Jun 5;9(1):8427. PMID: 30385841; PMCID: PMC6212531.
- [3]Maïga H, Lu D, Mamai W, Bimbilé Somda NS, Wallner T, Bakhoum MT, Bueno Masso O, Martina C, Kotla SS, Yamada H, Salvador Herranz G, Argiles Herrero R, Chong CS, Tan CH, Bouyer J. Standardization of the FAO/IAEA Flight Test for Quality Control of Sterile Mosquitoes. Front Bioeng Biotechnol. 2022 Jul 18;10:876675. doi: 10.3389/fbioe.2022.876675. PMID: 35923573; PMCID: PMC9341283.
- [4]Gómez, M.; Macedo, A.T.; Pedrosa, M.C.; Hohana, F.; Barros, V.; Pires, B.; Barbosa, L.; Brito, M.; Garziera, L.; Argilés-Herrero, R.; et al. Exploring Conditions for Handling Packing and Shipping *Aedes aegypti* Males to Support an SIT Field Project in Brazil. Insects 2022, 13, 871. https://doi.org/10.3390/insects13100871
- [5]Maïga H., Bakhoum M.T., Mamai W., G. Diouf, Bimbilé Somda N.S., Wallner T., Bueno Masso O., Martina C., Kotla S.S., Yamada H., Sow B.D.B., Fall A.G., and Bouyer J. From the lab to the field: Long-distance transport of sterile *Aedes* mosquitoes. Insects 2023, 14, x.

6. Acknowledgments

We are grateful to Maylen Gomez, Fabrizio Balestrino, Enkerlin Hoeflich Walther R, and Marc Vreysen for their useful comments/suggestions.

7. Appendix

Example of documents including no health hazard and no commercial value, that accompany the shipment.

Food and Agriculture
Organization of the
United Nations

IAEA
Atoms for Peace and Development

الوكالة الدولية للطاقة الذرية
国 际 原 子 能 机 构
International Atomic Energy Agency
Agence internationale de l'énergie atomique
Международное агентство по атомной энергии
Organismo Internacional de Energía Atómica

Joint FAO/IAEA Centre
of Nuclear Techniques in Food and Agriculture

Vienna International Centre, PO Box 100, 1400 Vienna, Austria
Phone: (+43 1) 2600 • Fax: (+43 1) 26007
Email: Official.Mail@iaea.org • Internet: http://www.iaea.org

In reply please refer to: F2606
Dial directly to extension: (+43 1) 2600-28274

2022-10-25

CERTIFICATE OF VALUE - ORIGIN OF GOODS

ORIGIN:<br><br><br><br><br><br>CONSIGNEE:	Insect Pest Control Laboratory<br>FAO/IAEA Centre of Nuclear Techniques in Food and Agriculture<br>IAEA Laboratories, Seibersdorf<br>Wagramer Straße 5, P.O. Box 100<br>A-1400 Vienna, Austria<br><br>Thierno Bakhoum<br>ISRA/LNERV,<br>Route du Front de Terre<br>Dakar<br><br>**SENEGAL**
MODE OF TRANSPORT:	**Courier - FedEx**
TOTAL VALUE:	EUR 1.00

All materials are non-hazardous, of no commercial value and used exclusively for scientific research.

Marc Vreysen
Laboratory Head
NAFA - Insect Pest Control Laboratory

Food and Agriculture
Organization of the
United Nations

IAEA

Atoms for Peace and Development

الوكالة الدولية للطاقة الذرية
国 际 原 子 能 机 构
International Atomic Energy Agency
Agence internationale de l'énergie atomique
Международное агентство по атомной энергии
Organismo Internacional de Energía Atómica

Joint FAO/IAEA Centre
of Nuclear Techniques in Food and Agriculture

Vienna International Centre, PO Box 100, 1400 Vienna, Austria
Phone: (+43 1) 2600 • Fax: (+43 1) 26007
E-mail: Official.Mail@iaea.org • Internet: http://www.iaea.org

In reply please refer to: F2606
Dial directly to extension: (+431) 2600-28259

2022-10-25

PROFORMA INVOICE

CONSIGNOR:	Insect Pest Control Laboratory<br>FAO/IAEA Centre of Nuclear Techniques in Food and Agriculture<br>IAEA Laboratories, Seibersdorf<br>Wagramer Straße 5, P.O. Box 100<br>A-1400 Vienna, Austria
CONSIGNEE:	Thierno Bakhoum<br>ISRA/LNERV,<br>Route du Front de Terre<br>Dakar<br><br>**SENEGAL**
DESCRIPTION:	The shipment contains sterile male adult mosquitoes
CONTAINER:	1 box
DECLARED AS:	Reference material
TOTAL VALUE:	EUR 1.00

Marc Vreysen
Laboratory Head
NAFA - Insect Pest Control Laboratory

Food and Agriculture
Organization of the
United Nations

IAEA

Atoms for Peace and Development

الوكالة الدولية للطاقة الذرية
国 际 原 子 能 机 构
International Atomic Energy Agency
Agence internationale de l'énergie atomique
Международное агентство по атомной энергии
Organismo Internacional de Energía Atómica

Joint FAO/IAEA Centre
of Nuclear Techniques in Food and Agriculture

Vienna International Centre, PO Box 100, 1400 Vienna, Austria
Phone: (+43 1) 2600 • Fax: (+43 1) 26007
E-mail: Official.Mail@iaea.org • Internet: http://www.iaea.org

In reply please refer to: F2606
Dial directly to extension: (+431) 2600-28259

2022-10-25

SANITARY CERTIFICATE

This is to certify that the sterile male adult mosquitoes included in this shipment are free of any parasites and diseases. The material has no commercial value, is not restricted and will be used exclusively for scientific research.

Marc Vreysen
Laboratory Head
NAFA - Insect Pest Control Laboratory

Food and Agriculture
Organization of the
United Nations

IAEA

Atoms for Peace and Development

الوكالة الدولية للطاقة الذرية
国 际 原 子 能 机 构
International Atomic Energy Agency
Agence internationale de l'énergie atomique
Международное агентство по атомной энергии
Organismo Internacional de Energía Atómica

Joint FAO/IAEA Division
of Nuclear Techniques in Food and Agriculture

Vienna International Centre, PO Box 100, 1400 Vienna, Austria
Phone: (+43 1) 2600 • Fax: (+43 1) 26007
E-mail: Official.Mail@iaea.org • Internet: http://www.iaea.org

In reply please refer to: F2606
Dial directly to extension: (+431) 2600-28259

PACKING LIST

DATE: 2022-10-25

CONSIGNOR:		Insect Pest Control Laboratory<br>FAO/IAEA Centre of Nuclear Techniques in Food and Agriculture<br>IAEA Laboratories, Seibersdorf<br>Wagramer Straße 5, P.O. Box 100<br>A-1400 Vienna, Austria
CONSIGNEE:		Thierno Bakhoum<br>ISRA/LNERV,<br>Route du Front de Terre<br>Dakar<br><br>**SENEGAL**
TRANSPORT	FROM:	Vienna, **AUSTRIA**
	TO:	Dakar, **SENEGAL**
MODE OF TRANSPORT:		Courier - Fedex
CONTENTS:		Sterile male adult mosquitoes
CONTAINER DESCRIPT		1 box
WEIGHT:		3 kg

SHIPPERS'S DECLARATION ABOUT GOODS FOR TRANSPORT
The shipper confirms and assures that the above-mentioned details about the goods for this transport are correct, complete and exact, and that the contents of the packed goods correspond to the real contents of the packages.

Marc Vreysen
Laboratory Head
NAFA - Insect Pest Control Laboratory

Food and Agriculture
Organization of the
United Nations

IAEA

Atoms for Peace and Development

الوكالة الدولية للطاقة الذرية
国 际 原 子 能 机 构
International Atomic Energy Agency
Agence internationale de l'énergie atomique
Международное агентство по атомной энергии
Organismo Internacional de Energía Atómica

Joint FAO/IAEA Centre
Nuclear Techniques in Food and Agriculture

Vienna International Centre, PO Box 100, 1400 Vienna, Austria
Phone: (+43 1) 2600 • Fax: (+43 1) 26007
E-mail: Official.Mail@iaea.org • Internet: http://www.iaea.org

In reply please refer to: F2606
Dial directly to extension: (+431) 2600-28259

2022-10-25

SUPPLIER STATEMENT

We declare that the value, for custom purposes, of the goods included in the shipment is EUR 1.00. The shipment includes sterile male adult mosquitoes for scientific research purposes.

Marc Vreysen
Laboratory Head
NAFA - Insect Pest Control Laboratory
