# Supplementary figures and images for: From the Lab to the Field: Long-Distance Transport of Sterile Aedes Mosquitoes
Source: Insects. 2023 Feb 18;14(2):207. doi: 10.3390/insects14020207 (PMC9967802; doi:10.3390/insects14020207)

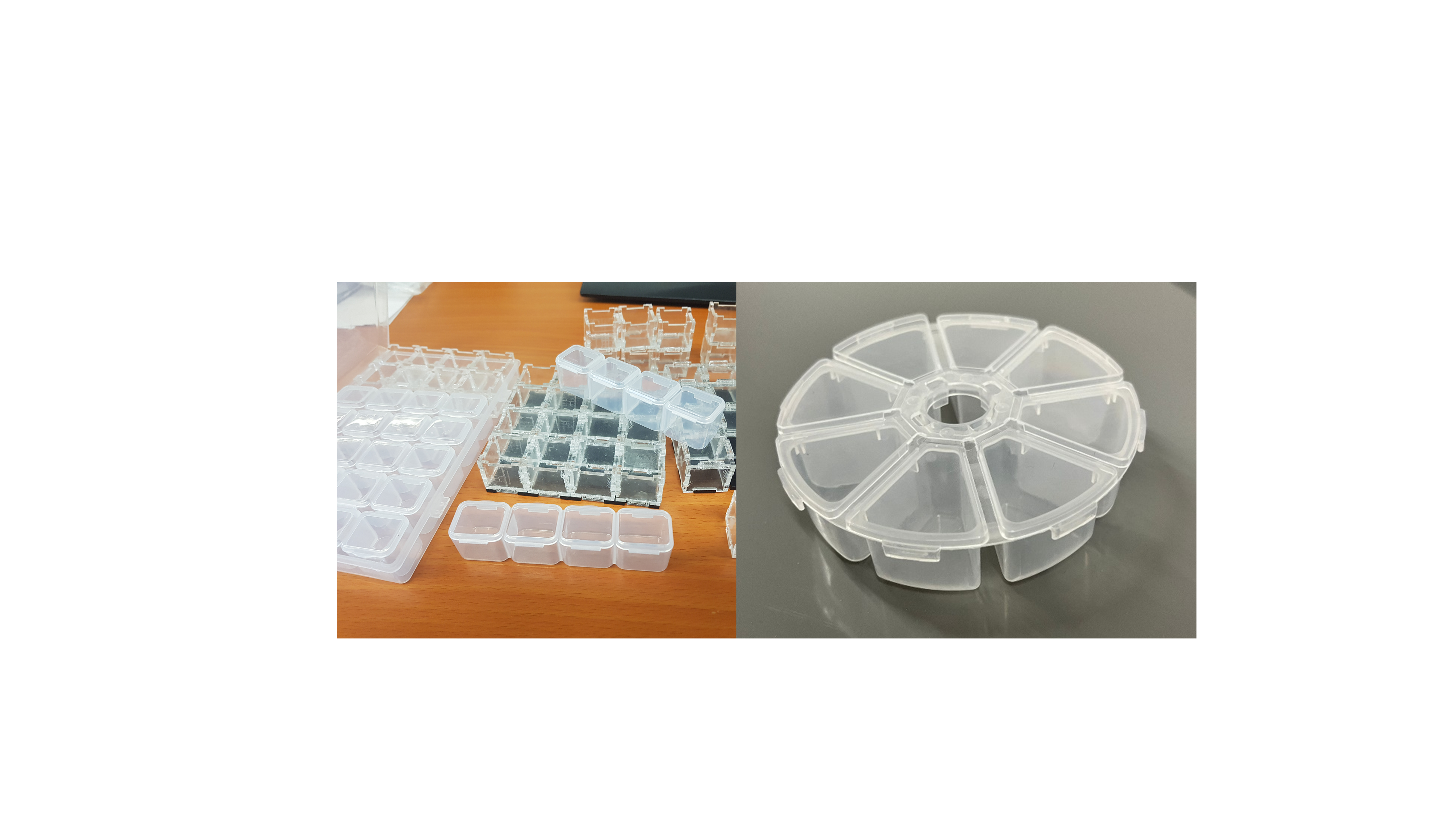

Supplement: Supplementary file 1 [file insects-14-00207-s001.zip › Supplementary File S2.tif]
